# Discrepancies in the diagnosis of hypertension in adolescents according to available office and home high blood pressure criteria

**DOI:** 10.1111/jch.14406

**Published:** 2021-12-09

**Authors:** Fabiana G. A. M. Feitosa, Audes D. M. Feitosa, Marco A. Mota‐Gomes, Annelise M. G. Paiva, Weimar S. Barroso, Roberto D. Miranda, Eduardo C. D. Barbosa, Andréa A. Brandão, Thiago S. V. Jardim, Paulo C. B. V. Jardim, Arthur B. M. Feitosa, Maria V. C. Santos, José L. Lima‐Filho, Andrei C. Sposito, Wilson Nadruz

**Affiliations:** ^1^ Laboratory of Immunopathology Keizo Asami Federal University of Pernambuco Recife PE Brazil; ^2^ Pronto Socorro Cardiológico de Pernambuco (PROCAPE) University of Pernambuco Recife PE Brazil; ^3^ University Hospital Oswaldo Cruz University of Pernambuco Recife PE Brazil; ^4^ UNICAP Clinical Research Institute Recife PE Brazil; ^5^ CESMAC University Center/Heart Hospital of Alagoas Maceió AL Brazil; ^6^ Hypertension League, Cardiovascular Section Federal University of Goiás Goiânia GO Brazil; ^7^ Cardiovascular Section, Geriatrics Division, Paulista School of Medicine Federal University of São Paulo São Paulo SP Brazil; ^8^ Hospital Israelita Albert Eistein São Paulo SP Brazil; ^9^ Department of Hypertension and Cardiometabolism São Francisco Hospital ‐ Santa Casa de Porto Alegre Porto Alegre Brazil; ^10^ School of Medical Sciences State University of Rio de Janeiro Rio de Janeiro RJ Brazil; ^11^ Department of Congenital Heart Disease and Pediatric Cardiology of the Brazilian Society of Cardiology Rio de Janeiro RJ Brazil; ^12^ Department of Internal Medicine School of Medical Sciences State University of Campinas SP Paulo Brazil

**Keywords:** adolescents, home blood pressure, hypertension, office blood pressure

## Abstract

This study aimed at comparing the prevalence of abnormal blood pressure (BP) phenotypes among 241 adolescents referred for hypertension (15.4 ± 1.4 years, 62% males, 40% obese) according to mostly used or available criteria for hypertension [AAP or ESH criteria for high office BP (OBP); Arsakeion or Goiânia schools’ criteria for high home BP monitoring (HBPM)]. High OBP prevalence was greater when defined by AAP compared with ESH criteria (43.5% vs. 24.5%; *p* < .001), while high HBPM prevalence was similar between Arsakeion and Goiânia criteria (33.5% and 37.5%; *p* = .34). Fifty‐five percent of the sample fulfilled at least one criterion for high BP, but only 31% of this subsample accomplished all four criteria. Regardless of the HBPM criteria, AAP thresholds were associated with lower prevalence of normotension and masked hypertension and greater prevalence of white‐coat and sustained hypertension than ESH thresholds. These findings support the need to standardize the definition of hypertension among adolescents.

## 1 INTRODUCTION

The diagnosis of hypertension in adolescents relies on office blood pressure (OBP) measures, and thresholds used to identify abnormal values are usually derived from guidelines of the European Society of Hypertension (ESH) and the American Academy of Pediatrics (AAP).^1^, ^2^ However, OBP thresholds recommended by these societies markedly differ, due to discrepancies in normative blood pressure (BP) tables and the age at which BP classification is replaced by adult classification.^3^

Evaluation of out‐of‐office BP in adolescents has been encouraged by current guidelines, aiming at unmasking white‐coat and masked BP effects and identifying the true hypertension phenotype.^1^, ^4^ Growing attention has been devoted to home BP monitoring (HBPM), because of its feasibility, good agreement with ambulatory BP monitoring (ABPM), and relatively low cost.^1^, ^5^ However, HBPM use in adolescents is still limited, and the prevalence of white‐coat hypertension (WH) and masked hypertension (MH) defined by HBPM, especially when comparing OBP thresholds defined by ESH and AAP, is uncertain. Furthermore, only two cross‐sectional studies suggested normalcy values for HBPM in adolescents,^6^, ^7^ but whether these normative data have similar ability to detect out‐of‐office hypertension is unknown. This study aimed at estimating the magnitude of the divergence in hypertension and BP phenotypes prevalence according to mostly used or available criteria to identify adolescents with high OBP and HBPM.

## 2 METHODS

We performed a cross‐sectional analysis of 241 adolescents with 12–17 years‐old from 129 Brazilian centers who were referred for evaluation of hypertension and performed HBPM from December, 2017 to April, 2021 using an online platform (www.telemrpa.com).^8^, ^9^, ^10^ The protocol was approved by the Oswaldo Cruz University Hospital/PROCAPE Complex Ethics Committee, which waived the requirement for informed consent. Data on age, sex and body mass index (BMI) were collected. OBP and HBPM were measured as previously reported,^8^, ^9^, ^10^ with the participants in the sitting position using appropriate cuff sizes and upper arm cuff devices (HEM‐7320 or HEM‐9200T; Omron Healthcare, Japan) validated by the American National Standards Institute, Inc/Association for the Advancement of Medical Instrumentation/International Organization for Standardization (ANSI/AAMI/ISO) 81060–2:2009 guidelines, and assumed to be suitable to evaluate BP among individuals over 12 years‐old.^11^ OBP was calculated as the average of two BP readings assessed after at least 3 min of rest. HBPM was calculated as the average of all home BP measurements (23.0 ± 2.3 readings) comprising three home BP measurements in the morning and in the evening after at least 3 min of rest for four consecutive days, before antihypertensive medications were taken. HBPM measures started on the next day after OBP measurements. Obesity and overweight were diagnosed based on World Health Organization criteria.^12^

As a primary analysis, we used two criteria to define hypertension based on OBP: (1) 2017 AAP criteria (AAP‐OBP) = values ≥95 percentile from normalcy tables in adolescents with 12–13 years‐old and ≥130/80 mm Hg in adolescents with 14–17 years‐old^2^; and (2) 2016 ESH criteria (ESH‐OBP) = values ≥95 percentile from normalcy tables in adolescents with 12–15 years‐old and ≥140/90 mm Hg in adolescents with 16–17 years‐old.^1^ Two criteria were used to define hypertension based on HBPM: (1) values ≥95 percentile from normalcy tables based on the Arsakeion School study, Greece (Arsakeion‐HBPM)^6^; and (2) values ≥95 percentile from normalcy tables based on the Goiânia schools study, Brazil (Goiânia‐HBPM),^7^ as long as the they were < 135/85 mm Hg.^2^ BP phenotypes were defined as: normotension (normal OBP and HBPM), WH (high OBP and normal HBPM), MH (normal OBP and high HBPM) and sustained hypertension (high OBP and HBPM).^1^, ^2^ As a secondary analysis, we evaluated the performance of reference OBP values derived from 73,999 Brazilian adolescents with 12–17 years‐old (Brazilian‐OBP) to define hypertension and BP phenotypes.^13^

Continuous and categorical variables are presented as mean±standard deviation and proportion. Comparisons of categorical and continuous variables were performed using chi‐square test and Student t‐test, respectively. *p*‐values < .05 were considered significant. Statistical analysis was performed using Stata software Version 14.1 (Stata Corp LP, College Station, TX, USA).

## 3 RESULTS

The sample (age = 15.4 ± 1.4 years, 62% males, 40% obese, 29% with overweight and 7% using antihypertensive medications) had BMI = 27.9 ± 6.9 kg/m^2^, height = 1.70 ± 0.09 m, office SBP = 114.7 ± 15.8 mm Hg, office DBP = 76.2 ± 10.9 mm Hg, home SBP = 113.6 ± 12.9 mm Hg and home DBP = 74.3 ± 8.8 mm Hg. Abnormal BP prevalence was greater when defined by AAP‐OBP compared with ESH‐OBP (43.5% vs. 24.5%; *p* < .001), but was similar between Arsakeion‐HBPM and Goiânia‐HBPM (33.5% and 37.5%; *p* = .34). Furthermore, adolescents with abnormal BP defined by all criteria had higher BMI, office BP and HBPM (Table S1).

**FIGURE 1:**
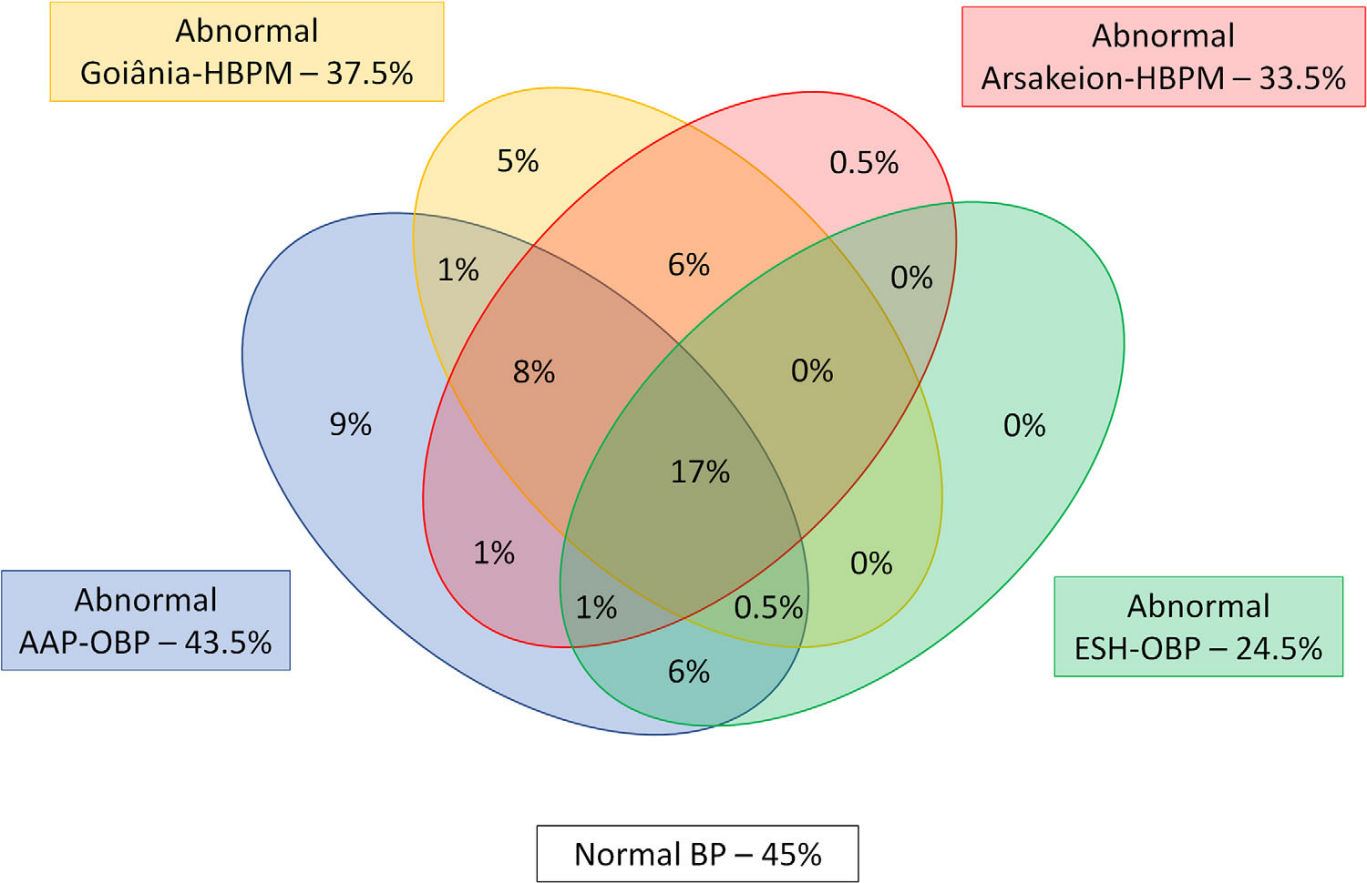
Venn diagram demonstrating the overlap of abnormal blood pressure prevalence according to the studied criteria. AAP‐OBP ‐ American Academy of Pediatrics high office blood pressure criteria; ESH‐OBP ‐ European Society of Hypertension high office blood pressure criteria; Arsakeion‐HBPM – Arsakeion school study high home blood pressure monitoring criteria; Goiânia‐HBPM – Goiânia schools study high home blood pressure monitoring criteria

The Venn diagram shown in Figure 1 presents the overlap of abnormal BP prevalence according to AAP‐OBP, ESH‐OBP, Arsakeion‐HBPM or Goiânia‐HBPM. Fifty‐five percent of the sample had at least one criterion for hypertension. Regarding OBP measures, 43.5% of the sample had hypertension defined by either AAP‐OBP or ESH‐OBP, with 24.5% and 19% having hypertension defined by both criteria and solely by AAP‐OBP, respectively. Regarding HBPM measures, 40% of the sample had hypertension defined by either Arsakeion‐HBPM or Goiânia‐HBPM, with 31%, 6.5% and 2.5% having abnormal BP defined by both criteria, and solely by Goiânia‐HBPM or Arsakeion‐HBPM, respectively. Importantly, only 17% of the sample had hypertension according to all studied criteria, which corresponded to 31% of individuals with at least one criterion for hypertension.

Regardless of the HBPM criteria, AAP‐OBP and ESH‐OBP had moderate/good agreement in the identification of hypertension phenotypes (Table S2), and AAP‐OBP thresholds were associated with lower prevalence of normotension and MH and greater prevalence of WH and sustained hypertension (Table 1). Furthermore, the sum of adolescents with WH and MH ranged between 22% and 28% when considering the possible combinations of the studied OBP and HBPM criteria.

**TABLE 1:** Hypertension phenotypes based on the combination of the studied criteria for the diagnosis of abnormal office and home blood pressure

Phenotype, %	AAP‐OBP and Arsakeion‐HBPM	AAP‐OBP and Goiânia‐HBPM	ESH‐OBP and Arsakeion‐HBPM	ESH‐OBP and Goiânia‐HBPM	*p*‐value^a^
Normotension	50	45	60	56	.009
White‐coat hypertension	17	17	7	7	<.001
Masked hypertension	6	11	15	20	<.001
Sustained hypertension	27	27	18	17	0009

*Abbreviations*: AAP‐OBP, American Academy of Pediatrics high office blood pressure criteria; ESH‐OBP, European Society of Hypertension high office blood pressure criteria; Arsakeion‐HBPM, Arsakeion school study high home blood pressure monitoring criteria; Goiânia‐HBPM, Goiânia schools study high home blood pressure monitoring criteria.

^a^ *p*‐values were estimated by chi‐square test.

Results of additional analysis showed that: (1) the prevalence of abnormal BP by Brazilian‐OBP was 43% and its performance to identify hypertension and BP phenotypes was more similar to AAP‐OBP than to ESH‐OBP (Tables S3 and S4, Figure S1); (2) obese participants had greater prevalence of high OBP and sustained hypertension, and AAP‐OBP was associated with higher rates of high OBP and sustained hypertension than ESH‐OBP, particularly among obese participants (Tables S5 and S6); and (3) high HBPM prevalence did not change when using the first two home BP readings rather than triplicate measurements on each occasion (Table S7).

## 4 DISCUSSION

This study confirmed that the identification of high OBP was greater when using AAP‐OBP compared with ESH‐OBP,^3^ and provided novel evidence that identification of high HBPM was similar between Arsakeion‐HBPM and Goiânia‐HBPM. Given that Arsakeion‐HBPM and Goiânia‐HBPM data were derived from Greek and Brazilian populations, respectively, it can be suggested that the combination of AAP‐OBP and Goiânia‐HBPM may be more appropriate for evaluation of non‐European adolescents. Conversely, Brazilian‐OBP had similar performance to identify hypertension and BP phenotypes compared to AAP‐OBP, and might be an attractive alternative approach for the diagnosis of hypertension among Brazilian adolescents.

WH and MH are associated with higher prevalence of hypertensive organ‐damage in adolescents, and presumably higher cardiovascular risk later in life than normotension.^1^, ^4^, ^14^ In our analysis, approximately one fourth of the participants had WH or MH independent of the criteria for abnormal OBP and HBPM. This prevalence is similar to that of adolescents referred to alternative hypertension centers for elevated BP, but greater than that of school samples, which would be more representative of general populations.^5^, ^15^ Additionally, the rates of WH and MH were greater and lower, respectively, when using AAP‐OBP compared with ESH‐OBP, regardless of the HBPM criteria, which agrees with ABPM data obtained in a large alternative sample of adolescents.^3^

This study has limitations. First, OBP was estimated from two readings obtained with oscillometric devices after at least 3 min of rest, while current guidelines usually recommend assessment of three OBP readings obtained by the auscultatory method after 5 min of rest.^1^, ^2^ Additionally, we used triplicate morning and evening HBPM measurements for four consecutive days, while ESH guidelines recommend two measurements per occasion preferably on seven consecutive days.^1^ Together, these discrepancies might have influenced the prevalence of hypertension phenotypes in our study. Second, the BP devices, albeit validated by ANSI/AAMI/ISO guidelines, have not consistently included adolescents in their validations.

In conclusions, we found marked discrepancies in the prevalence of abnormal BP phenotypes when applying the mostly used criteria for high OBP and HBPM in adolescents. These data support the need for standardization of hypertension definition among adolescents.

## AUTHOR CONTRIBUTIONS

F.G.A.M.F., A.D.M.F. and W.N.J. conceived and designed the study, analyzed the data, interpreted results and drafted the manuscript. M.A.M.‐G., A.M.G.P., W.S.B., R.D.M., E.C.D.B., A.A.B., T.S.V.J., P.C.B.V.J., A.B.M.F., M.V.C.S., J.L.L.‐F. and A.C.S. analyzed the data, interpreted results and edited and revised the manuscript. All gave final approval and agree to be accountable for all aspects of work ensuring integrity and accuracy.

## CONFLICTS OF INTEREST

A.D.M.F., M.A.M.‐G., W.S.B., A.A.B., R.D.M. and E.C.D.B. are owners of the online TELEMRPA platform (Beliva, Brazil). A.D.M.F., M.A.M.‐G. and W.S.B. are consultants for Omron.

## Supporting information

Supporting Information
