# Supplementary material for: Discrepancies in the diagnosis of hypertension in adolescents according to available office and home high blood pressure criteria
Source: J Clin Hypertens (Greenwich). 2021 Dec 9;24(1):83–7. doi: 10.1111/jch.14406 (PMC8783324; doi:10.1111/jch.14406)
Supplement: Supplementary file 1 — Supporting Information [file JCH-24-83-s001.docx]

**SUPPLEMENTAL MATERIAL**

**Supplemental Table 1.** Characteristics of the participants according to the studied criteria for the diagnosis of abnormal office and home blood pressure

| Variables | AAP-OBP | | ESH-OBP | | Arsakeion-HBPM | | Goiânia-HBPM | |
| --- | --- | --- | --- | --- | --- | --- | --- | --- |
|  | Normal BP | Abnormal BP | Normal BP | Abnormal BP | Normal BP | Abnormal BP | Normal BP | Abnormal BP |
| N | 136 | 105 | 182 | 59 | 161 | 80 | 151 | 90 |
| Male sex, % | 58 | 68 | 59 | 71 | 63 | 61 | 64 | 59 |
| Age, years | 15.4±1.4 | 15.3±1.5 | 15.5±1.4 | 14.9±1.4† | 15.3±1.5 | 15.5±1.4 | 15.4±1.5 | 15.3±1.4 |
| Weight, kg | 76.0±19.0 | 86.6±24.5‡ | 78.9±20.9 | 85.7±25.1* | 77.2±19.9 | 87.4±24.9‡ | 77.2±20.4 | 86.3±23.9† |
| Height, m | 1.69±0.09 | 1.70±0.10 | 1.69±0.09 | 1.70±0.09 | 1.70±0.09 | 1.69±0.09 | 1.70±0.09 | 1.68±0.09 |
| Body mass index, kg/m^2^ | 26.5±6.2 | 29.7±7.5‡ | 27.4±6.6 | 29.5±7.7* | 26.6±6.2 | 30.6±7.6‡ | 26.4±6.1 | 30.4±7.5‡ |
| Obesity, % | 31 | 52† | 37 | 51 | 32 | 58† | 31 | 57‡ |
| Overweight, % | 33 | 23 | 30 | 24 | 33 | 20* | 34 | 20* |
| Office SBP, mmHg | 106.6±11.4 | 125.1±14.5‡ | 110.2±13.2 | 128.7±14.8‡ | 110.8±14.4 | 122.6±15.6‡ | 111.0±14.6 | 120.8±15.9‡ |
| Office DBP, mmHg | 68.9±6.1 | 85.6±8.2‡ | 72.1±8.0 | 88.7±9.1‡ | 71.7±9.0 | 85.1±8.9‡ | 72.0±9.4 | 83.1±9.8‡ |
| Home SBP, mmHg | 109.2±11.8 | 119.2±12.2‡ | 111.2±12.2 | 121.1±12.4‡ | 109.3±11.3 | 122.4±11.5‡ | 108.8±11.2 | 121.6±11.7‡ |
| Home DBP, mmHg | 70.3±7.0 | 79.5±8.3‡ | 71.8±7.3 | 81.9±8.9‡ | 69.7±5.5 | 83.5±6.9‡ | 69.4±5.8 | 82.3±7.3‡ |
| Antihypertensive med, % | 6 | 9 | 6 | 10 | 5 | 11 | 5 | 11 |

BP – blood pressure; DBP – diastolic blood pressure; SBP – systolic blood pressure; med - medications

AAP-OBP - American Academy of Pediatrics high office BP criteria; ESH-OBP - European Society of Hypertension high office BP criteria; Arsakeion-HBPM – Arsakeion school study high home BP monitoring criteria; Goiânia-HBPM – Goiânia schools study high home BP monitoring criteria

* p<0.05; † p<0.01; ‡ p<0.001 compared with normal BP within the same criteria.

**Supplemental Table 2.** Agreement (kappa statistic) between AAP-OBP and ESH-OBP in the identification of hypertension phenotypes

| Hypertension phenotypes | Prevalence derived from AAP-OBP | Prevalence derived from ESH-OBP | Kappa |
| --- | --- | --- | --- |
| *Derived from Arsakeion-HBPM* |  |  |  |
| Normotension | 50% | 60% | 0.80 |
| White-coat hypertension | 17% | 7% | 0.53 |
| Masked hypertension | 6% | 15% | 0.54 |
| Sustained hypertension | 27% | 18% | 0.74 |
| *Derived from Goiânia-HBPM* |  |  |  |
| Normotension | 45% | 56% | 0.80 |
| White-coat hypertension | 17% | 7% | 0.54 |
| Masked hypertension | 11% | 20% | 0.65 |
| Sustained hypertension | 27% | 17% | 0.74 |

AAP-OBP - American Academy of Pediatrics high office blood pressure criteria; ESH-OBP - European Society of Hypertension high office blood pressure criteria; Arsakeion-HBPM – Arsakeion school study high home blood pressure monitoring criteria; Goiânia-HBPM – Goiânia schools study high home blood pressure monitoring criteria

**Supplemental Table 3.** Characteristics of the participants according to Brazilian-OBP for the diagnosis of abnormal office blood pressure

| Variables | Normal BP | Abnormal BP |
| --- | --- | --- |
| N | 138 | 103 |
| Male sex, % | 61 | 64 |
| Age, years | 15.5±1.4 | 15.2±1.5 |
| Weight, kg | 77.5 ± 20.0 | 84.8 ± 24.2* |
| Height, m | 1.70 ± 0.09 | 1.69 ± 0.09 |
| Body mass index, kg/m^2^ | 26.6 ± 6.2 | 29.7 ± 7.5‡ |
| Obesity, % | 33 | 51† |
| Overweight, % | 32 | 24 |
| Office SBP, mmHg | 107.7 ± 12.6 | 124.0 ± 14.7‡ |
| Office DBP, mmHg | 69.0 ± 6.2 | 85.8 ± 8.2‡ |
| Home SBP, mmHg | 109.7 ± 12.1 | 118.7 ± 12.3‡ |
| Home DBP, mmHg | 70.4 ± 7.1 | 79.5 ± 8.2‡ |
| Antihypertensive med, % | 6 | 9 |

Brazilian-OBP ­–Brazilian office blood pressure criteria; BP – blood pressure; DBP – diastolic blood pressure; SBP – systolic blood pressure; med - medications

* p<0.05; † p<0.01; ‡ p<0.001 compared with normal BP.

**Supplemental Table 4.** Hypertension phenotypes based on the combination of Brazilian-OBP, Arsakeion-HBPM and Goiânia-HBPM

| Phenotype, % | Brazilian-OBP and  Arsakeion-HBPM | Brazilian -OBP and  Goiânia-HBPM | p-value |
| --- | --- | --- | --- |
| Normotension | 51 | 47 | 0.11 |
| White-coat hypertension | 16 | 15 | 0.90 |
| Masked hypertension | 6 | 10 | 0.13 |
| Sustained hypertension | 27 | 28 | 0.92 |

Brazilian-OBP - Brazilian office blood pressure criteria; Arsakeion-HBPM – Arsakeion school study high home blood pressure monitoring criteria; Goiânia-HBPM – Goiânia schools study high home blood pressure monitoring criteria


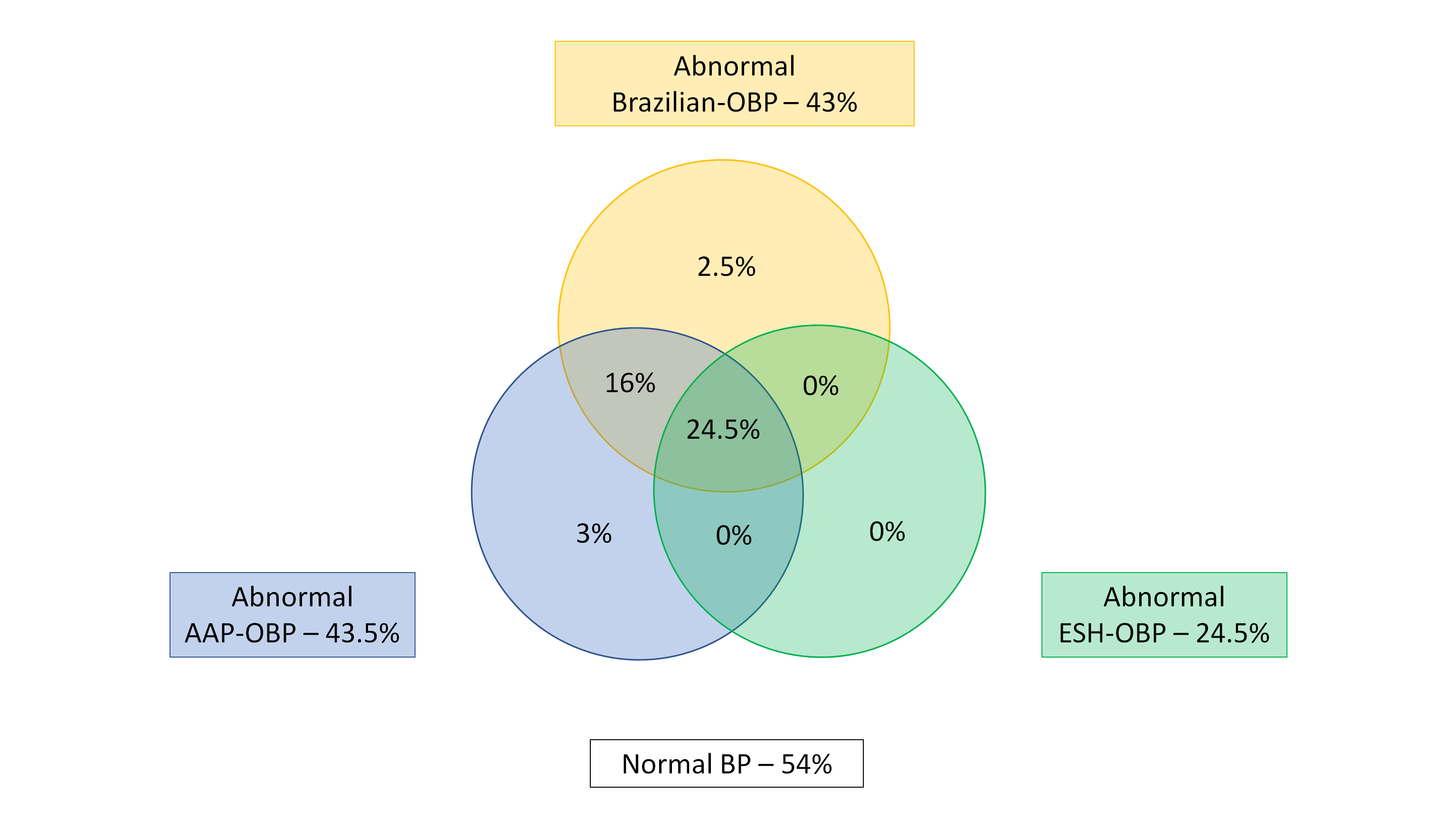


**Supplemental Figure 1. Venn diagram demonstrating the overlap of abnormal blood pressure prevalence according to the studied office blood pressure criteria.**

AAP-OBP - American Academy of Pediatrics high office blood pressure criteria; ESH-OBP - European Society of Hypertension high office blood pressure criteria; Brazilian-OBP - Brazilian office blood pressure criteria.

**Supplemental Table 5.** Prevalence of high BP in non-obese and obese participants

| Hypertension criteria | Prevalence of high BP, % | | p-value |
| --- | --- | --- | --- |
|  | Non-obese (n=144) | Obese (n=97) |  |
| AAP-OBP | 35 | 57 | <0.001 |
| ESH-OBP | 20 | 31 | 0.06 |
| Brazilian-OBP | 35.5 | 53.5 | 0.005 |
| Arsakeion-HBPM | 23.5 | 47.5 | <0.001 |
| Goiânia-HBPM | 27 | 52.5 | <0.001 |

AAP-OBP - American Academy of Pediatrics high office BP criteria; ESH-OBP - European Society of Hypertension high office BP criteria; Brazilian-OBP ­– Brazilian office blood pressure criteria; Arsakeion-HBPM – Arsakeion school study high home BP monitoring criteria; Goiânia-HBPM – Goiânia schools study high home BP monitoring criteria

**Supplemental Table 6.** Prevalence of hypertension phenotypes (in percentage) based on the combination of the studied criteria for the diagnosis of abnormal office and home blood pressure in non-obese and obese participants.

| Variables | AAP-OBP | | ESH-OBP | | Brazilian-OBP | |
| --- | --- | --- | --- | --- | --- | --- |
|  | Non-obese  (n=144) | Obese  (n=97) | Non-obese  (n=144) | Obese  (n=97) | Non-obese  (n=144) | Obese  (n=97) |
| *Derived from Arsakeion-HBPM* |  |  |  |  |  |  |
| Normotension | 60 | 36* | 68 | 47* | 60 | 38* |
| White-coat hypertension | 16 | 17 | 8 | 5 | 16 | 15 |
| Masked hypertension | 6 | 7 | 11 | 22* | 5 | 7 |
| Sustained hypertension | 18 | 40* | 13 | 26* | 19 | 40* |
| *Derived from Goiânia-HBPM* |  |  |  |  |  |  |
| Normotension | 57 | 29* | 66 | 40* | 58 | 32* |
| White-coat hypertension | 16 | 19 | 7 | 7 | 15 | 16 |
| Masked hypertension | 8 | 14 | 14 | 29* | 7 | 14 |
| Sustained hypertension | 19 | 38* | 13 | 24* | 20 | 38* |

BP – blood pressure; DBP – diastolic blood pressure; SBP – systolic blood pressure; med - medications

AAP-OBP - American Academy of Pediatrics high office BP criteria; ESH-OBP - European Society of Hypertension high office BP criteria; Brazilian-OBP ­– Brazilian office blood pressure criteria; Arsakeion-HBPM – Arsakeion school study high home BP monitoring criteria; Goiânia-HBPM – Goiânia schools study high home BP monitoring criteria

* p<0.05 compared with non-obese within the same OBP and HBPM criteria.

**Supplemental Table 7.** HBPM values and prevalence of high HBPM using the first 2 home BP readings or triplicate home BP readings on each occasion

| Variables | First 2 readings  per occasion | Triplicate readings per occasion | P |
| --- | --- | --- | --- |
| *HBPM values* |  |  |  |
| Systolic BP, mmHg | 114.1±12.9 | 113.6±12.9 | 0.68 |
| Diastolic BP, mmHg | 74.7±8.8 | 74.3±8.8 | 0.63 |
| *Prevalence of high HBPM* |  |  |  |
| Arsakeion-HBPM, % | 33.5 | 33.5 | 0.92 |
| Goiânia-HBPM, % | 37.5 | 37.5 | 1.00 |

BP – blood pressure; HBPM – home BP monitoring; Arsakeion-HBPM – Arsakeion school study high home BP monitoring criteria; Goiânia-HBPM – Goiânia schools study high home BP monitoring criteria
